# Molecular Basis of Resistance to Muramidase and Cationic Antimicrobial Peptide Activity of Lysozyme in Staphylococci

**DOI:** 10.1371/journal.ppat.0030102

**Published:** 2007-07-27

**Authors:** Silvia Herbert, Agnieszka Bera, Christiane Nerz, Dirk Kraus, Andreas Peschel, Christiane Goerke, Michael Meehl, Ambrose Cheung, Friedrich Götz

**Affiliations:** 1 Microbial Genetics Department, University of Tübingen, Tübingen, Germany; 2 Medical Microbiology and Hygiene Department, University of Tübingen, Tübingen, Germany; 3 Department of Microbiology, Dartmouth Medical School, Hanover, New Hampshire, United States of America; University of Pennsylvania, United States of America

## Abstract

It has been shown recently that modification of peptidoglycan by *O*-acetylation renders pathogenic staphylococci resistant to the muramidase activity of lysozyme. Here, we show that a Staphylococcus aureus double mutant defective in *O*-acetyltransferase A (OatA), and the glycopeptide resistance-associated two-component system, GraRS, is much more sensitive to lysozyme than S. aureus with the *oat*A mutation alone. The *gra*RS single mutant was resistant to the muramidase activity of lysozyme, but was sensitive to cationic antimicrobial peptides (CAMPs) such as the human lysozyme-derived peptide _107_R-A-W-V-A-W-R-N-R_115_ (LP9), polymyxin B, or gallidermin. A comparative transcriptome analysis of wild type and the *gra*RS mutant revealed that GraRS controls 248 genes. It up-regulates global regulators (*rot, sar*S, or *mgr*A), various colonization factors, and exotoxin-encoding genes, as well as the *ica* and *dlt* operons. A pronounced decrease in the expression of the latter two operons explains why the *gra*RS mutant is also biofilm-negative. The decrease of the *dlt* transcript in the *gra*RS mutant correlates with a 46.7% decrease in the content of esterified d-alanyl groups in teichoic acids. The *oat*A*/dlt*A double mutant showed the highest sensitivity to lysozyme; this mutant completely lacks teichoic acid–bound d-alanine esters, which are responsible for the increased susceptibility to CAMPs and peptidoglycan *O*-acetylation. Our results demonstrate that resistance to lysozyme can be dissected into genes mediating resistance to its muramidase activity (*oat*A) and genes mediating resistance to CAMPs *(gra*RS and *dlt)*. The two lysozyme activities act synergistically, as the *oat*A*/dlt*A or *oat*A*/gra*RS double mutants are much more susceptible to lysozyme than each of the single mutants.

## Author Summary

In humans, lysozyme plays an important role in the suppression of bacterial infections. However, some bacterial pathogens, such as *Staphylococcus aureus,* are completely resistant to lysozyme. Here we demonstrate that lysozyme acts on S. aureus in two ways: as a muramidase (cell wall lytic enzyme) and as a cationic antimicrobial peptide (CAMP). S. aureus has developed resistance mechanisms against both activities by modifying distinct cell wall structures. Modification of the peptidoglycan by *O*-acetylation (OatA) renders the cells resistant to the muramidase activity. Modification of teichoic acids by d-alanine esterification (Dlt) renders the cells resistant to lysozyme's CAMPs and other CAMPs. Transcriptome analysis of the glycopeptide resistance-associated (GraRS) two-component system revealed that this global regulator controls 248 genes such as other global regulators, colonization factors, or exotoxin-encoding genes. Since GraRS also upregulates the *dlt* operon, it was not surprising that in the *gra*RS mutant teichoic acid d-alanylation is markedly decreased, which explains its increased sensitivity to CAMPs. By comparative analysis of mutants we were able to dissect genes that were responsive to the dual activities of lysozyme. Here we show how efficiently S. aureus is protected from the human defense system, which enables this pathogen to cause persistent infections.

## Introduction

In humans, lysozyme is found in a wide variety of fluids, such as tears, breast milk, and respiratory and saliva secretions, as well as in cells of the innate immune system, including neutrophils, monocytes, macrophages, and epithelial cells [1,2]. Lysozyme is an important protein in the innate defense response against invading microorganisms and acts on bacteria by hydrolyzing the ß-1,4 glycosidic bonds between *N*-acetylmuramic acid (MurNAc) and *N*-acetylglucosamine (GlucNAc), resulting in degradation of peptidoglycan (PG), and subsequent cell lysis [3,4]. Most bacterial species are sensitive to lysozyme, but some important human pathogens, such as *Staphylococcus aureus, Neisseria gonorrhoeae,* and *Proteus mirabilis,* are resistant. The mechanisms behind the high resistance of S. aureus to lysozyme are unknown, although several studies suggest that *O*-acetylation at position *C*-6 of the MurNAc residue contributes to lysozyme resistance [5–9]. Recently, we were able to prove that indeed *O*-acetyltransferase A (OatA) of S. aureus is responsible for *O*-acetylation of the PG, and this leads to resistance to the muramidase activity of lysozyme [10]. We also showed that the MurNAc was *O*-acetylated only in pathogenic, lysozyme-resistant staphylococci (e.g., *S. aureus, S. epidermidis, S. lugdunensis,* and others). All nonpathogenic species (e.g., *S. carnosus, S. gallinarum,* or S. xylosus) were lysozyme sensitive and lacked PG-specific *O*-acetylation. Therefore, OatA can be regarded as a general virulence factor [11].

Although the *oat*A mutant was less resistant to lysozyme than the wild type (WT) *S. aureus,* it still was more resistant than, for example, *Micrococcus luteus,* suggesting that other factors, such as a high degree of peptide cross-linking, may also contribute to lysozyme resistance [12]. Recently, we showed that the presence of wall teichoic acid (WTA) increased lysozyme resistance [13]. One also has to consider that lysozyme does not only comprise muramidase activity but also antimicrobial peptide activity, as demonstrated by catalytically inactivate lysozyme or peptides isolated from digested lysozyme, and by synthetic lysozyme-derived peptides [14–17].

Here, we show that the extremely high resistance of S. aureus to lysozyme can be genetically dissected as a) resistance to muramidase activity and b) resistance to inherent cationic antimicrobial peptide (CAMP) activity. Furthermore, we characterized via transcriptome analysis the two-component system (TCS), GraRS, which, in addition to many virulence genes, also controls the *dlt* operon to mediate resistance to lysozyme and other CAMPs.

## Results

### Susceptibility of *oat*A and *gra*RS Single and Double Mutants to Lysozyme and CAMPs

**Figure 1:**
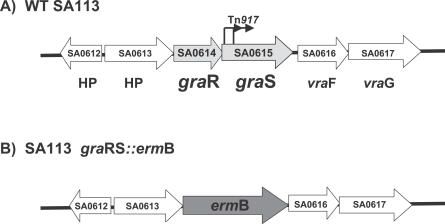
Illustration of Construction of the *gra*RS Deletion Mutant (A) Gene organization in the chromosome of WT SA113; Tn*917* insertions in *gra*S gene are indicated by arrows. (B) In the *gra*RS deletion mutant, *gra*RS is substituted by the erythromycin B resistance cassette. Note that *erm*B gene has a weak transcription terminator, and transcriptional read-through to the following *vra*FG genes is likely. *gra*R, response regulator; *gra*S, sensor histidine kinase; *vra*F, ABC transporter ATP-binding protein; *vra*G, ABC transporter permease; SA0612 and SA0613 are hypothetical proteins (HP).

**Figure 2:**
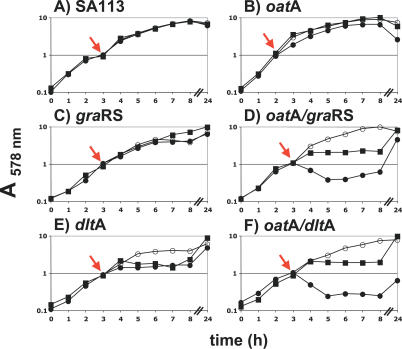
Susceptibility of WT SA113 and Various S. aureus Mutants to Lysozyme and Heat-Inactivated Lysozyme Cells were grown in BM at 37 °C. OD_578nm_ was measured hourly for the first 8 h and after 24 h. Lys was added in the exponential growth phase at OD_578nm_ 1.0 as indicated by arrow. Catalytic inactive Lys was heated for 1 h at 100 °C. (A) WT SA113: control (○); Lys (300 μg/ml [20.8 μM]) (•); heat-inactivated lysozyme (Lys) (300 μg/ml [20.8 μM]) (▪). (B) *oat*A mutant: control (○); Lys (300 μg/ml) (•); heat-inactivated Lys (300 μg/ml) (▪). (C) *gra*RS mutant: control (○); Lys (300 μg/ml) (•); heat-inactivated Lys (300 μg/ml) (▪). (D) *oat*A/*gra*RS mutant: control (○); Lys (50 μg/ml [3.47 μM]) (•); heat-inactivated Lys (300 μg/ml) (▪). (E) *dlt*A mutant: control (○); Lys (300 μg/ml) (•); heat-inactivated Lys (300 μg/ml) (▪). (F) *oat*A/*dlt*A mutant: control (○); Lys (20 μg/ml [1.39 μM]) (•); heat-inactivated Lys (300 μg/ml) (▪).

In our search for highly susceptible lysozyme mutants in *S. aureus,* we isolated two Tn*917* transposon mutants in SA113*oat*A*::kan* that revealed higher sensitivity to lysozyme than the *oat*A mutation alone. Chromosomal sequencing of the flanking Tn*917* insertion sites revealed that Tn*917* was inserted in SA0615 [18]. SA0615 and the upstream gene SA0614 have the features of a typical TCS and were recently named GraRS (glycopeptide resistance-associated), because overexpression of GraR (response regulator) and GraS (sensor histidine kinase) increased vancomycin resistance [19]. To further study the role of TCS in lysozyme resistance, we constructed a deletion mutant by substituting *gra*RS with an erythromycin B cassette to yield SA113 *gra*RS*::erm* (Figure 1). In addition, we also constructed an *oat*A*::kan/gra*RS*::erm* double knockout. Sequencing and complementation with pTX*gra*RS, a vector in which the *gra*RS genes are induced into expression by xylose, confirmed the correct replacement. Whereas the *oat*A*/gra*RS double mutant was highly susceptible to lysozyme, both single mutants were only marginally affected, but were still more sensitive than the WT, which is completely lysozyme resistant (Figure 2A–2D).

**Figure 3:**
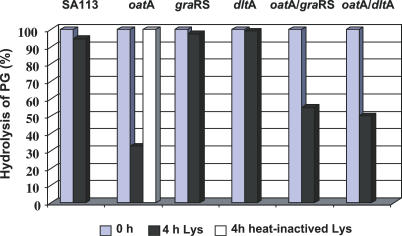
Susceptibility of PG Isolated from WT and Various Mutants to Hydrolysis by Lysozyme PG (0.5 mg/ml) isolated from WT SA113 and mutants were incubated with catalytic active lysozyme (Lys); in addition, the *oat*A mutant was incubated with heat-inactivated Lys (300 μg/ml) in 80 mM sodium phosphate-buffered saline. Lysis of PG was measured as a decrease in OD_660nm_ and calculated in percent. The diagram shows 100% of PG in the beginning (0 h) and the remaining undigested PG after 4 h of lysozyme treatment.

**Figure 4:**
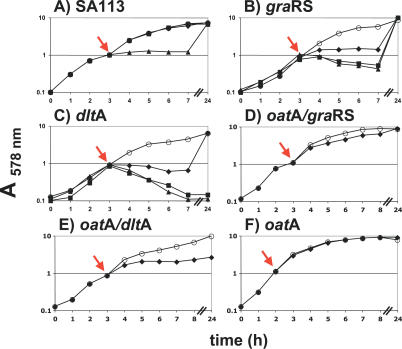
Susceptibility of WT and Various S. aureus Mutants to CAMPs (A) WT SA113: control (○); LP9 (200 μg/ml [164.9 μM]) (♦); polymyxin B (PMB) (20 μg/ml [14.4 μM]) (); and gallidermin (Gdm) (8 μg/ml [3.64 μM]) (▴). (B) *gra*RS mutant: control (○); LP9 (200 μg/ml) (♦); PMB (20 μg/ml) (); and Gdm (8 μg/ml) (▴). (C) *dlt*A mutant: control (○); LP9 (200 μg/ml) (♦); PMB (20 μg/ml) (); and Gdm (8 μg/ml) (▴). (D) *oat*A/*gra*RS mutant: control (○); LP9 (200 μg/ml) (♦). (E) *oat*A/*dlt*A mutant: control (○); LP9 (200 μg/ml) (♦). (F) *oat*A mutant: control (○); LP9 (200 μg/ml) (♦). Cells were grown in BM at 37 °C. OD_578nm_ was measured hourly for the first 7–8 h and after 24 h. CAMPs were added in the exponential growth phase at OD_578nm_ 1.0 as indicated by arrow.

The *oat*A*/gra*RS double mutant was much more lysozyme sensitive than each of the single mutants. This hypersensitivity of the double mutant can be explained by dual activities of lysozyme that act in a synergistic way. To study this phenotype in more detail, we investigated whether the *gra*RS single mutant is affected by the muramidase activity of lysozyme. Indeed, the isolated PG from the *gra*RS single mutant was completely resistant to lysozyme hydrolysis, in contrast to the *oat*A mutant. As expected, PG of the *oat*A/*gra*RS double mutant was also hydrolysed, although the sensitivity was less pronounced, as in the *oat*A single mutant (Figure 3). Therefore, the increased sensitivity of the double mutant likely came from its higher susceptibility to lysozyme's CAMP activity. This was confirmed by the addition of LP9, polymyxin B, or gallidermin to a growing culture, which caused immediate growth arrest in the *gra*RS mutant, whereas the WT was much less affected (Figure 4A and 4B), and only the lantibiotic gallidermin inhibited the WT. In addition, we demonstrated that heat-inactivated lysozyme exhibits CAMP activity, but no muramidase activity. Heat-inactivated lysozyme showed no activity (neither lytic nor CAMP activity) to the *oat*A mutant or to the isolated PG of *oat*A, but it was able to inhibit the growth of the *oat*A/*gra*RS double mutant (Figures 2B, 2D, and 3). This result suggests that GraRS controls genes involved in CAMP resistance. This effect was not only achieved with hen egg-white, but also with human lysozyme.

### Comparative Transcriptome Analysis of WT and *gra*RS Mutant

To find out which genes are responsible for the high susceptibility to CAMPs in the *gra*RS mutant, we carried out a comparative transcriptome analysis of the WT strain and the *gra*RS mutant. We detected 115 genes whose mRNAs were up-regulated (Table 1) and 133 genes whose mRNAs were down-regulated by GraRS (Table 2). The complete list of up- and down-regulated genes with their National Center for Biotechnology Information PID numbers is presented in Dataset S1. In order to give an impression of which genes are controlled by GraRS, some examples are mentioned below.

**Table 1:**
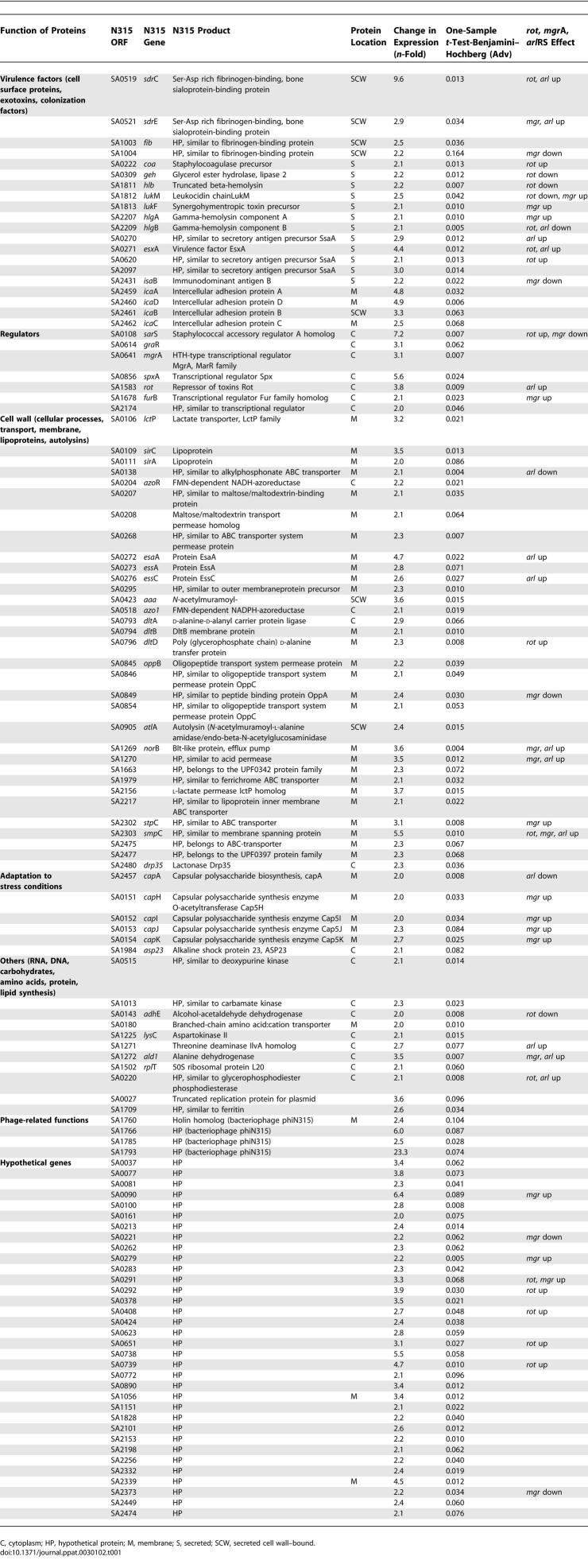
115 S. aureus SA113 Genes Up-Regulated by GraRS

**Table 2:**
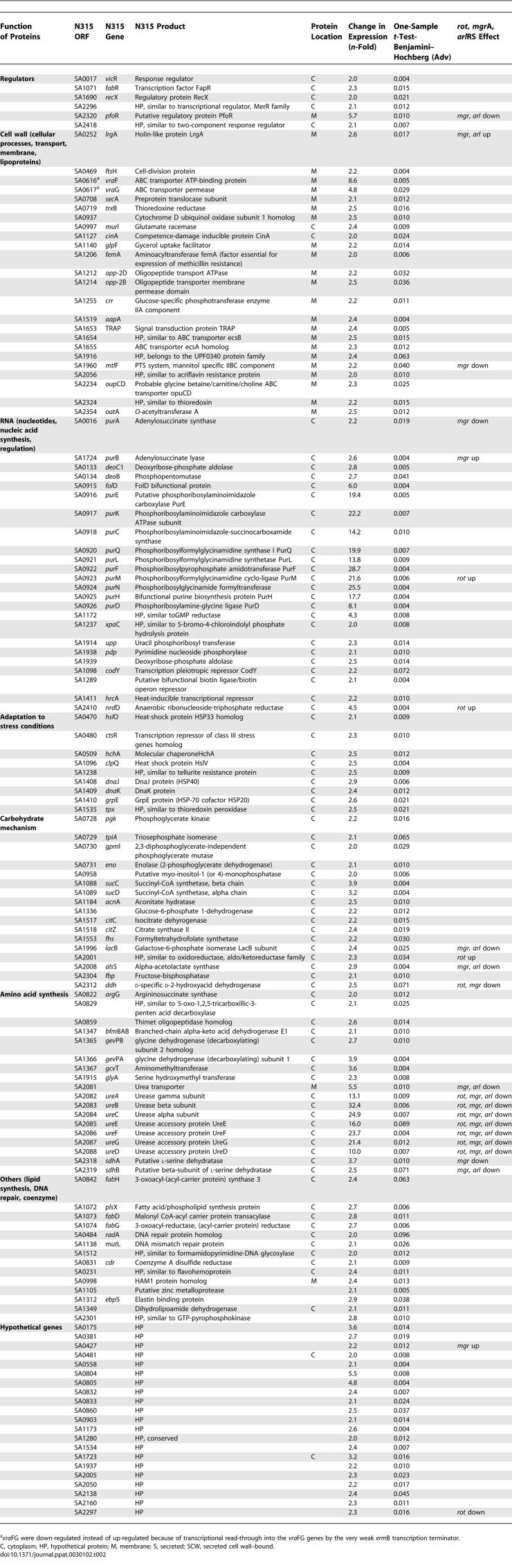
133 S. aureus SA113 Genes Down-Regulated by GraRS

In the *gra*RS mutant, genes that are involved in RNA and amino acid synthesis and glycolysis shows highly gene transcription rates. In particular, the urease genes (*ure*A-G) all 12 *pur* genes were 2- to 32-fold up-regulated as compared to the WT, whereas *pur*R (repressor) appeared not to be influenced by GraRS. Interestingly, the amount of *oat*A transcript increased in the *gra*RS mutant, which could explain the slightly higher resistance of the *gra*RS mutant to the muramidase activity of lysozyme (Figure 3). A number of genes that were down-regulated included global regulators (*rot, sar*S*, mgr*A), cell surface protein encoding genes (the Ser-Asp rich fibrinogen-binding proteins SdrC and SdrE), the major autolysin gene (*atl*A) and an autolysin/adhesin gene *(aaa)* [20], exoprotein encoding genes (*hlb, hlg*A,B, *luk*M,F, and *geh*), transporter encoding genes (*ess*A/*ess*C, *opp*B, and *nor*B), capsule encoding genes (*cap*A,H,I,J,K) and PIA encoding genes (*ica*ADBC), genes responsible for d-alanyl esterification of teichoic acids (TAs) (*dlt*A,B,D), and the alanine dehydrogenase gene (*ald*1). The pronounced decrease of expression of the *ica* [21–23] and *dlt* operons [24] and *atl*A [25] explains why the *gra*RS mutant showed a biofilm-negative phenotype on microtiter plates (unpublished data). With a few genes, such as *rot, ure*C, and *dlt*A, we verified the transcriptome data by reverse transcriptase (RT)-PCR (Table 3).

**Table 3:**
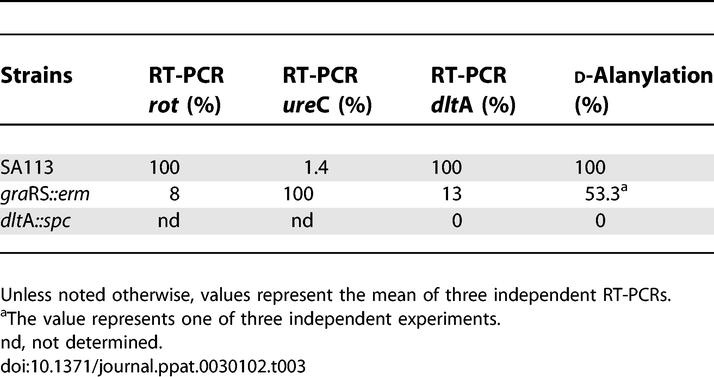
RT-PCR Values and d-Alanylation of TAs

Next, we asked which of the 115 less expressed genes in the *gra*RS mutant were responsible for the increased susceptibility to CAMPs. A most likely candidate was the *dlt* operon (encoding enzymes involved in d-alanylation of TAs). Its transcript was decreased 2.1-fold to 2.9-fold as compared to WT, and indeed, the d-alanylation of TAs was decreased 46.7% in the *gra*RS mutant compared to WT (Table 3). It has been previously shown that inactivation of the *dlt* operon in S. aureus confers sensitivity to defensins, protegrins, and other antimicrobial peptides [26]. The observed decrease of *ald*1 transcription by a factor of 3.5 is in line with the decreased *dlt* transcription. Ald1 is the alanine dehydrogenase, which is involved in the synthesis of l-alanine.

### Comparison of *gra*RS and *dlt*A Mutants

Because the *dlt* operon is less expressed in the *gra*RS mutant, we investigated lysozyme susceptibility with a *dlt*A deletion mutant, which is well-known to be sensitive to CAMPs [26]. Indeed, the *dlt*A mutant was more sensitive to lysozyme (Figure 2E); however, this sensitivity was not due to its muramidase activity, as the isolated PG of the *dlt*A mutant was not hydrolyzed by lysozyme (Figure 3). Furthermore, growth of the *dlt*A mutant was inhibited whether active or heat-inactivated lysozyme was applied (Figure 2E). When the susceptibility of *gra*RS and *dlt*A mutants to LP9, polymyxin B, and gallidermin were compared, both mutants were similarly more susceptible to these CAMPs (Figure 4B and 4C). However, there were two distinctions: a) the susceptibility of the *dlt*A mutant was more pronounced than that of the *gra*RS mutant, and b) even in the presence of gallidermin or polymyxin B, the *gra*RS mutant started to grow after some time and reached the same optical density (OD) values after 24 h as the control culture lacking CAMPs. In contrast, the *dlt*A mutant remained sensitive to gallidermin and polymyxin B and was unable to resume growth. In the presence of LP9, growth resumed after a similar lag period as in the *gra*RS mutant; this can possibly be explained by its proteolytic degradation. Not only the single but also the double mutants *oat*A/*gra*RS and *oat*A/*dlt*A were sensitive to the CAMP activity of LP9, although the susceptibility was less pronounced as with the *gra*RS and *dlt*A single mutants. However, the *oat*A single mutant was completely resistant to LP9, indicating that *oat*A is resistant to CAMPs (Figure 4D–4F). With respect to gallidermin- and polymyxin B–induced cell lysis, it has been observed that CAMPs such as lantibiotics induce autolysis in staphylococci by increasing PG hydrolase activity [27]. We assume that gallidermin and polymyxin B, which are also CAMPs, very likely have a similar effect.

**Figure 5:**
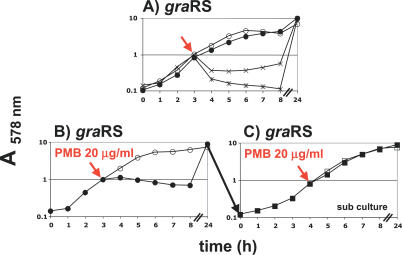
Susceptibility of *S. aureus gra*RS Mutant to Mutanolysin, Mutanolysin and LP9 or Lysozyme, and Polymyxin B (A) *gra*RS mutant: control (○); mutanolysin (Mut) (100 μg/ml [4.35 μM]) (•); Mut (100 μg/ml) and LP9 (200 μg/ml) (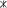); Mut (50 μg/ml [2.18 μM]) and Lys (300 μg/ml) (×). (B) *gra*RS mutant: control (○); polymyxin B (PMB) (20 μg/ml) (•). (C) *gra*RS subculture of 5B: control (□); PMB (20 μg/ml) (▪). Cells were grown in BM at 37 °C. OD_578nm_ was measured hourly for the first 8 h and after 24 h. Cationic agents were added in the exponential growth phase at OD_578nm_ 1.0 as indicated by arrow.

We asked whether the increasing insensitivity of the *gra*RS mutant after prolonged growth is some short lasting CAMP-induced adaptation or whether it is based on selection of resistant mutants. To answer this question, we inoculated from a 24-h *gra*RS culture treated with polymyxin B (Figure 5B) a new culture and challenged it again with polymyxin B (Figure 5C). The subculture revealed no growth retardation, which suggests that the *gra*RS phenotype is unstable and that polymyxin B–resistant revertants were quickly selected. Since the *dlt*A revealed a stable phenotype, we assume that in the selected revertants *dlt*A expression was increased to WT levels.

### Hypersensitivity of the *oat*A*/dlt*A and *oat*A/*gra*RS Double Mutants to Lysozyme

The highest susceptibility to lysozyme was observed with the *oat*A*/dlt*A double mutant, which was more than 66-fold and 333-fold more sensitive to lysozyme than the *dlt*A and *oat*A single mutants, respectively (Figure 2B, 2E, and 2F; Table 4). The *oat*A/*gra*RS mutant is not quite as sensitive as the *oat*A*/dlt*A mutant. Another difference is that the *oat*A*/dlt*A mutant stays lysozyme sensitive even after 24 h of cultivation (Figure 2D and 2F), indicating that the *dlt*A mutant phenotype cannot easily revert to the WT phenotype. The lower susceptibility of the *oat*A/*gra*RS double mutant can possibly be explained by the fact that the TA in this mutant still contains 53.3% d-alanyl esters, whereas the *dlt*A mutant completely lacks d-alanylation in its TAs (Table 3).

**Table 4:**
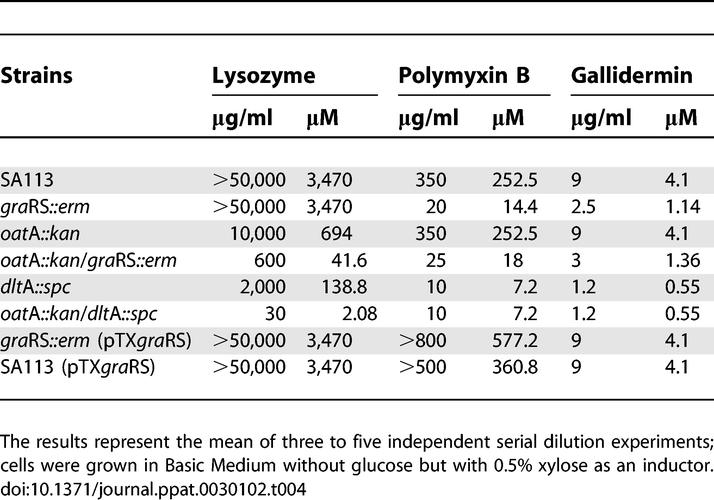
MIC Values of SA113 and Various Mutants

The high susceptibility of the double mutants is based on the dual activities of lysozyme: a) the *oat*A mutant is sensitive to the muramidase activity of lysozyme but is insensitive to CAMPs (Figures 2B, 3, and 4F), and b) the *dlt*A and *gra*RS mutants are sensitive to CAMPs, but insensitive to the muramidase activity of lysozyme (Figures 3, 4B, and 4C). The extremely high lysozyme susceptibility of the *oat*A*/dlt*A double mutant can only be explained by a synergistic effect of the two activities.

### Increased Lytic Activity of Mutanolysin by Lysozyme and LP9 in the *gra*RS Mutant

Mutanolysin is a muramidase that is able to hydrolyze *O*-acetylated PG [28] but does not normally cause cell lysis in WT S. aureus or its *gra*RS mutant at a concentration of 100 μg/ml. However, when the *gra*RS mutant was treated with mutanolysin in combination with lysozyme or LP9, the lytic activity (indicated by decrease in OD) was strongly increased (Figure 5A). Because the *O*-acetylated *gra*RS mutant is insensitive to the catalytic activity of lysozyme, we assume that mutanolysin acts through its lytic activity, and LP9 and lysozyme through their CAMP properties. We have not investigated how the stimulating effect of lysozyme and LP9 on cell lysis is accomplished. However, we assume that it is caused by the concerted action of PG hydrolysis by mutanolysin and induced autolysis by lysozyme and LP9, as mentioned above.

### Minimal Inhibition Concentration Values of SA113 and Various Mutants

The minimal inhibition concentration (MIC) values for lysozyme, polymyxin B, and gallidermin in WT and various mutants are summarized in Table 4. Both the WT and the *gra*RS mutant were completely resistant to lysozyme at a concentration of 50 mg/ml. However, the *gra*RS mutant was 17- and 4-fold more susceptible to polymyxin B or gallidermin. The sensitivity to the CAMPs is very likely due to the aforementioned decrease in expression of the *dlt* operon, which corresponds with decreased d-alanylation of the TAs. The *oat*A mutant was more susceptible to lysozyme than the *gra*RS mutant, but, similar to WT, was completely insensitive to heat-inactivated lysozyme or CAMPs, indicating that *oat*A is only sensitive to the muramidase activity of lysozyme. The *oat*A*/gra*RS double mutant was almost 17-fold more sensitive to lysozyme than the *oat*A mutant, which can be explained by the fact that this double mutant is sensitive to both the muramidase and the CAMP activities of lysozyme. The two activities exert a synergistic effect on the double mutant. The *dlt*A single mutant was over 25-fold more sensitive to lysozyme than the WT and 5-fold more sensitive than the *oat*A single mutant, demonstrating the importance of lysozyme's CAMP activity. Furthermore, the *dlt*A mutant exhibited the highest susceptibility to polymyxin B and gallidermin, but was completely insensitive to lysozyme's muramidase activity (Figure 3).

With a MIC of only 30 μg/ml, the *oat*A*/dlt*A double mutant revealed the highest susceptibility to lysozyme. Indeed, it has a 20-fold greater sensitivity to lysozyme than the *oat*A*/gra*RS double mutant. The *oat*A*/dlt*A double mutant is 333-fold and 66-fold more sensitive than the single *oat*A or *dlt*A mutants, which illustrates the extremely high synergistic effect of lysozyme when it can exert both muramidase and CAMP activities. Overexpression of *gra*RS in the *gra*RS mutant or the WT by pTX*gra*RS resulted in an approximately 2-fold increase in polymyxin B resistance, indicating that even in WT cells, CAMP resistance can be further increased.

## Discussion

One of our research aims was to identify genes involved in staphylococcal lysozyme resistance. We have already elucidated two genes and corresponding enzymes that contribute to resistance against the muramidase activity of lysozyme. Since the target of muramidase is PG, it is not surprising that the mechanism of resistance is masking PG by modification. In S. aureus there are two PG modifications that are involved in resistance to lysozyme's muramidase activity. One modification is *O*-acetylation catalyzed by the PG-specific *O*-acetyltransferase A, OatA, and we have shown that the *oat*A mutant is more susceptible to the muramidase activity of lysozyme than the WT [10]. The other modification is WTA [29] that is covalently linked to the same *C_6_* position in MurNAc as in the *O*-acetyl group. TagO is a specific UDP-*N*-acetylglucoseamine transferase, which is involved in the first step of WTA synthesis. The *tag*O deletion mutant completely lacks WTA [30]. Although the *tag*O mutant still shows high lysozyme resistance, a *oat*A*/tag*O double mutant, however, is much more susceptible to lysozyme's muramidase activity than the *oat*A mutation alone [13]. Here, we show that the high lysozyme resistance of S. aureus is not only based on resistance to the muramidase activity of lysozyme, but also to its inherent CAMP resistance.

The described global two-component regulator, GraRS, was identified in an *oat*A-minus background by increased lysozyme susceptibility in an *oat*A*/gra*RS double mutant. The *gra*RS mutant was more susceptible to CAMPs than the WT. We assume that the reason for the increased susceptibility of the *gra*RS mutant was a decrease in *dlt* expression, and consequently, GraRS up-regulates *dlt* expression. The Dlt enzymes modify TAs by the incorporation of d-alanine esters rendering the cells resistant to CAMPs, very likely by repulsion [26]. We showed that the *dlt*A mutant is even more susceptible to lysozyme-derived LP9 and other CAMPs than the *gra*RS mutant, because in the *dlt*A mutant, d-alanine esters were completely absent in TAs, the mutant was stable, and no revertants were observed. Heat-inactivated lysozyme does not affect either the growth of the *oat*A or that of the *gra*RS mutant. The latter effect is surprising, as the *gra*RS mutant is sensitive to the other CAMPs (LP9, gallidermin, polymyxin B). However, the *oat*A/*gra*RS mutant was sensitive to heat-inactivated lysozyme, suggesting that the bulky molecule has better access to the cell envelope when the PG is de-*O*-acetylated. Likewise, sensitivity of the *dlt*A mutant to heat-inactivated lysozyme can also be explained by better access to the cell envelope because of the lack of d-alanine esters in TAs.

The next interesting question was, how do CAMPs act in the *dlt*A, *oat*A/*gra*RS, or *oat*A/*dlt*A mutants? Killing of Gram-negative bacteria could be demonstrated by lysozyme-derived peptides that were transported through the outer membrane and damaged the inner membrane by pore formation [17]. Several authors assume that lysozyme and CAMPs are not only acting as membrane permeabilization agents, but also activate autolytic wall enzymes of Gram-positive bacteria, thus causing cell lysis [31–33]. It has also been shown that lipoteichoic acids can bind and inhibit autolysins, depending on their degree of d-alanylation [34–36]. Similar results were also obtained in a *dlt* mutant of *Lactococcus lactis,* which showed increased autolysis [37]. In line with these observations, the *gra*RS and *dlt*A mutants also showed increased autolysis when treated with Triton X-100 (unpublished data), suggesting that in these mutants, too, CAMPs activate autolytic enzymes. We assume that the observed synergistic effect of lysozyme in the *oat*A/*gra*RS and *oat*A/*dlt*A double mutants is caused by the simultaneous activation of autolytic enzymes and the muramidase activity of lysozyme. A similar synergistic effect is seen by treatment with mutanolysin in combination with LP9 (inducing autolysis) or lysozyme (cannot exert its muramidase activity as the PG is *O*-acetylated) as shown in the *gra*RS single mutant (Figure 5A). For the first time (to our knowledge), we have traced and dissected genes that were responsive to the dual activities of lysozyme.

Until now, little was known about the two-component system GraRS. We became interested in the regulation of GraRS because we wanted to trace the gene(s) that caused the increased CAMP susceptibility in the *gra*RS mutant. Comparative transcriptome analysis of SA113, an 8325-derivative, and its *gra*RS mutant revealed that 115 genes were up-regulated and 133 genes were down-regulated by GraRS (Tables 1 and 2). Among the down-regulated genes was the *vra*FG operon, which immediately follows the *gra*RS operon. However, in studying intermediate level of vancomycin resistance in *S. aureus,* Ambrose Cheung and colleagues found that *vra*FG is positively controlled by GraRS [38]. This contradictory result can be explained by the genetic organization of our *gra*RS*::erm*B deletion mutant (Figure 1). In our mutant, the *erm*B cassette is in the same orientation as the *vra*FG genes. Since the *erm*B transcription terminator is very weak, we assume that there is a transcriptional read-through into the *vra*FG genes. This explains why in our *gra*RS deletion mutant, the *vra*FG genes were up-regulated instead of down-regulated.

**Figure 6:**
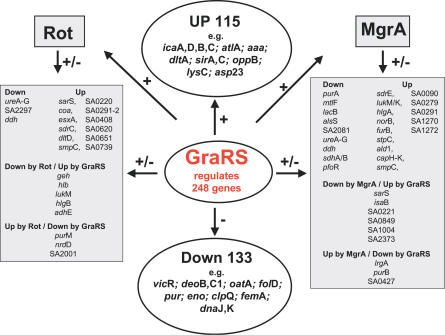
Interplay of GraRS–TCS with Other Global Regulators Of the 248 genes regulated by GraRS, 115 genes are up-regulated and 133 genes are down-regulated. GraRS also upregulates the global regulators Rot and Mgr (both are homologs of SarA). Genes that are controlled by both GraRS and Rot or GraRS and MgrA are boxed. Example genes that are exclusively controlled by GraRS are circled.

GraRS up-regulates transcription of global regulators such as the SarA homologs Rot, SarS, and MgrA. We compared our GraRS transcriptome results with that of the recently published transcriptome studies of Rot [39], MgrA [40], and ArlRS [41] (Tables 1 and 2; Figure 6). Rot is a repressor of exoproteins but positively regulates cell surface proteins, and SarS is a positive activator of protein A. MgrA appears to be an antagonist to Rot, as it up-regulates exoproteins and down-regulates cell surface proteins, including the regulator SarS. We found that Rot and MgrA regulate some of the GraRS-controlled genes in the same direction. For these few genes we do not know whether their up- or down-regulation is directly affected by GraRS or indirectly via up-regulation of Rot and MgrA, respectively. Moreover, there are some genes that were regulated in opposite directions (Figure 6, boxed genes). Interestingly, GraRS up-regulates both regulators, Rot 3.8- and MgrA 3.1-fold. GraRS controls many genes involved in cell wall synthesis and transport (57 genes). Among the transporters are the EssA and EssC proteins, involved in transport of the virulence factor EsxA, oligopeptide transport system (OppB), or NorB, which encodes the Blt-like protein that is an efflux pump involved in multidrug resistance, all of which are up-regulated by GraRS. Interestingly, *smp*C, which encodes a membrane-spanning protein with unknown transport functions, is the only gene that is increased by all four regulators (GraRS, Rot, MgrA, and ArlRS). The gene which had the highest (23.3-fold) up-regulation by GraRS was SA1793, which encodes a hypothetical protein with a phage-related function. Many of the down-regulated genes are involved in RNA and amino acid synthesis or glycolysis. *lrg*A, which encodes a holin-like protein with murein hydrolase activity, is also down-regulated by GraRS but up-regulated by ArlRS and MgrA. Most of the genes are exclusively regulated by GraRS, such as *ica, pur, mgr*A, *sir*A,C, *atl*A, *aaa, dna*J,K, *grp*E, and *vra*F,G. These results illustrate that there is a distinct cross-regulation between GraRS, ArlRS, Rot, MgrA, and probably some other global regulators.

GraRS is not only important for resistance to glycopeptides, lysozyme, and other CAMPs. Our data suggest that GraRS also has an intermediate role between other global regulators (Agr, MgrA, Rot, and SarA), as GraRS up-regulates both adhesins as well as exoproteins and toxins (e.g., *hlb, hlg*A,B, *luk*M,F, *geh*). GraRS is possibly involved in the establishment of persistent infections by the up-regulation of colonization factors (e.g., *ica, atl, aaa, fib, sir*A, *sir*C, *sdr*C, *sdr*E), factors involved in resistance to CAMPs *(dlt),* factors involved in intermediary vancomycin resistance (*vra*F,G, as mentioned above), and factors involved in biofilm formation (e.g., *dlt, atl, ica*). It would be interesting to study the *gra*RS mutant in an animal model for chronic infection.

## Materials and Methods

### Bacterial strains and plasmids.

All of the strains and plasmids that were used are listed in Table 5. Bacteria were grown in Basic Medium (BM) (1% tryptone; Gibco BRL Life-Technologies, http://www.invitrogen.com/), 0.5% yeast extract (Gibco BRL), 0.5% NaCl, 0.1% K_2_HPO_4_, 0.1% glucose, or 0.5% xylose).

**Table 5:**
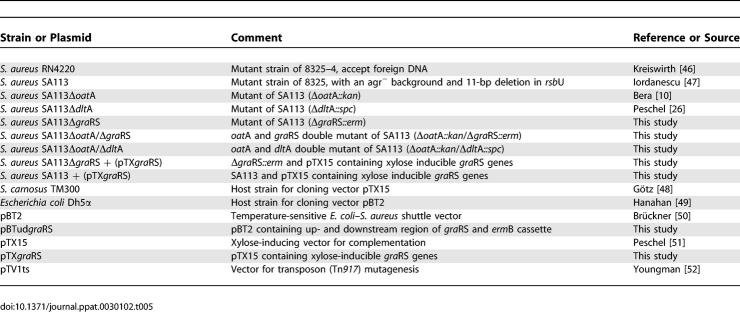
List of Strains and Plasmids

### Transposon mutagenesis.

Transposon mutagenesis was carried out in the Δ*oat*A*::kan* mutant using the temperature-sensitive plasmid pTV1ts and was performed as described by Bera et al. [10].

### Construction of plasmids, homologous recombination, and transduction.

Was performed as described by Bera et al. [10]. The PCR products, up- and downstream of *gra*RS (SA0614/15) (U0614/15Kpn: TGATATAGGTACCTAATTGTTTACTAGCCGACG, U0614/15Sma: ATTTGTCCCGGGTTCTAGTAGTATTTGCATCC, D0614/15Sal: GGCCGTGTCGACTTTGTCATTTTAAACATGCG, and D0614/15Nhe: ATTGCTAGCTTGGCATAACTTGCTGCAACAGG), were cloned into the polylinker of the pBT2 vector flanking the *erm*B antibiotic cassette. Complementation of the *gra*RS deletion mutant was obtained by cloning the *gra*RS genes (1,912 bp) (C0614/15Bam: AATGATGGATCCTGGCTTTGAAGTTGACTGCC, and C0614/15Eco: AGCGCGAATTCATTTCCTTTAGGCTTTGGCAC) into the xylose-inducible vector pTX15 in S. carnosus TM300. The *oat*A*::kan/dlt*A*::spc* double mutant was created by bacteriophage φ11-mediated transduction of the *oat*A*::kan* knockout into the *dlt*A*::spc* deletion mutant.

### Effects of cationic agents on exponential growth.

Overnight cultures were diluted to an OD_578nm_ of 0.1 in 50 ml of BM and the cultures were incubated with shaking at 37 °C. OD was determined every hour. Ten milliliters of each culture were transferred into a new 100-ml flask when the cultures reached an OD_578nm_ of nearly 1.0. Then, cationic agents, such as hen egg-white lysozyme and human lysozyme (Sigma-Aldrich, http://www.sigmaaldrich.com/), LP9 (a lysozyme-derived 9-aa peptide, _107_R-A-W-V-A-W-R-N-R_115_–NH_2_) (EMC, http://www.microcollections.de/), polymyxin B (Sigma-Aldrich), gallidermin (Genmedics, http://www.genmedics.com/), or mutanolysin (Sigma), were added. Lysozyme was inactivated by heating for 1 h at 100 °C and placed on ice. The OD_578nm_ of all cultures was measured hourly up to 7–8 h and after 24 h.

### MIC assay.

The overnight cultures were diluted in BM with 0.5% xylose to a concentration of 0.5 × 10^6^ CFU per ml and aliquoted in 0.5-ml samples, and cationic agents in different concentrations were added. The cultures were incubated with shaking at 37 °C for 20–24 h and MIC was determined.

### Biofilm assay.

An overnight culture was diluted 1:200 in fresh TSB with 0.5% glucose, and 200 μl were filled into microtiter plates and incubated for 20–24 h at 37 °C without shaking. The supernatant was removed and the plate was washed two times with PBS (pH 7.4). The plate was dried and the cells were colored with 0.1% safranine.

### Isolation of PG.

One liter of BM was inoculated with an overnight culture of the WT SA113 or the mutants. Strains were grown for 12 h with shaking at 37 °C. Cells were centrifuged, washed two times with cold 0.9% NaCl, diluted in 0.9% NaCl, and boiled for 20 min. After the cells were chilled on ice, they were again centrifuged and washed twice with 0.9% NaCl. The cells were disrupted in a mechanical grinding device using glass beads ∅150–212 μm (Sigma-Aldrich) at 4 °C, centrifuged and washed two times with cold H_2_O_bidest_, boiled for 30 min in 2% SDS to remove noncovalently bound proteins, and washed four times with H_2_O_bidest_. The cell wall fragments were diluted in 0.1 M Tris/HCl (pH 6.8) and incubated with 0.5 mg/ml trypsin for 16 h at 37 °C to degrade cell-bound proteins. After centrifugation and washing with water, the PG was lyophilized.

### Turbidometric assay of PG.

For analyzing the susceptibility of PG to lysozyme, we used a modified method turbidometric assay as described by Clarke [42]. The PG of the WT SA113 and the mutants were sonicated and diluted to 0.5 mg in 1 ml of 80 mM PBS (pH 6.4). After the addition of 300 μg lysozyme per ml, the decrease in optical density was monitored at the beginning (0 h) and after 4 h at OD_660nm_ and calculated as percentages.

### Quantification of d-alanylation of TA by HPLC.

S. aureus strains were grown in BM with 0.25% glucose overnight, centrifuged, washed three times, and resuspended in ammonium acetate buffer (20 mM [pH 6.0]). The OD_600nm_ was adjusted to 30. Aliquots (1 ml) were heat-inactivated by incubation at 99 °C for 10 min and centrifuged, and pellets were dried. After incubation at 37 °C for 1 h with 100 μl of 0.1 N NaOH, 100 μl of 0.1 N HCl were added for neutralization and samples were dried. For derivatization, 100 μl of triethylamine and 100 μl of Marfey's reagent (1-fluoro-2,4-dinitrophenyl-5-l-alanine amide; Sigma) (10 mM) were added. After incubation at 40 °C for 1 h, samples were dried and resuspended in DMSO:H_2_O (1:1). Quantification of d-alanine was performed by HPLC as previously described [43].

### RNA isolation and real-time RT-PCR.

SA113 and the *gra*RS deletion mutant were cultivated in 50 ml of BM and harvested at mid-exponential growth phase. Before RNA isolation, two volumes of RNAprotect bacteria reagent (Qiagen, http://www.qiagen.com/) were added to 10 ml of culture and centrifuged. The cells were lysed by the addition of 50 μg/ml of lysostaphin (0.5 mg/ml) (Genmedics) in TE buffer and total RNA was isolated using the RNeasy Mini Kit (Qiagen). Contaminating DNA was degraded with the DNase Kit (Ambion, http://www.ambion.com/) according to the manufacturer's instructions. LightCycler RT-PCR was carried out using the LightCycler RNA amplification Kit SYBR Green I or with the LightCycler RNA amplification kit for hybridization probes (Roche Biochemicals, http://www.roche.com/). The internal control *gyr* was quantified using 10-fold serial dilutions (10^4^ to 10^8^ copies/μl) of a specific RNA standard using oligonucleotides specific for *gyr* (gyr297F: TTAGTGTGGGAAATTGTCGATAAT and gyr574R: AGTCTTGTGACAATGCGTT TACA), *dlt*A (dltA1: TGGCGTTGAAAGACTAGGC and dltA2: TTACGAACTCAGACTGGCG), *rot* (rot1: TTCAGCGAGATTGAAAGCG and rot2: GTTGCTCTACTTGCAATGG) or *ure*C (ureC1: GATATCATTGCCGCTGAAGG and ureC2: AAAGCAGATGGTGTTGCACC) as described [44]. Standard curves for *dlt*A and *rot* were generated using 5-fold serial dilutions of WT SA113 RNA or for *ure*C of the *gra*RS mutant RNA. Differences between WT and the *gra*RS mutant were determined by *n*-fold change and calculated as a percentage of the mRNA product. The specificity of the PCR was verified by size determination of the amplicons by agarose gel electrophoresis. To check for DNA contamination, each sample was subjected to PCR by using the LightCycler DNA amplification kit SYBR Green I (Roche Biochemicals). In none of the cases an amplification product was detectable.

### Transcriptome analysis.

Transcriptome analysis was carried out as described by the microarray manufacturer Scienion (http://www.scienion.de/) and Resch et al. [45]. cDNA was synthesized from isolated RNA (1 μg) during mid-exponential growth (4 h) derived from WT SA113 (labeled in green with Cy3 [532 nm]) or from the *gra*RS mutant (labeled in red with Cy5 [635 nm]). cDNAs from WT and the *gra*RS mutant were pooled and hybridized on four DNA microarrays. Scienion performed DNA transcriptome analysis by comparing the intensity of each Cy3-labeled gene of the WT with the intensity of each Cy5-labeled gene of the *gra*RS mutant as a ratio of the medians (532/635). The threshold was set at a 2-fold difference in gene expression. Genes whose RNA level was higher in WT (2.0 and more) were categorized as being positively regulated by GraRS. In contrast, genes that had higher RNA levels (2.0 and more) in the *gra*RS mutant were described as being negatively regulated by GraRS. The significance of differences (*n*-fold) in gene expression was calculated by One-Sample *t*-Test-Benjamini–Hochberg (Adv); results <0.051 are significant, and some genes from Tables 1 and 2 are higher than 0.05.

## Supporting Information

Dataset S1 GraRS-Regulated Genes (36 KB XLS)

## Abbreviations

CAMP: cationic antimicrobial peptide
GraRS: glycopeptide resistance-associated
MIC: minimal inhibition concentration
MurNAc: *N*-acetylmuramic acid
OatA: *O*-acetyltransferase A
OD: optical density
PG: peptidoglycan
TA: teichoic acid
TCS: two-component system
WT: wild type
WTA: wall teichoic acid

## References

[ppat-0030102-b001] Jolles P, Jolles J (1984). What's new in lysozyme research? Always a model system, today as yesterday. Mol Cell Biochem.

[ppat-0030102-b002] Levy O (2000). Antimicrobial proteins and peptides of blood: Templates for novel antimicrobial agents. Blood.

[ppat-0030102-b003] Phillips D (1966). The three-dimensional structure of an enzyme molecule. Sci Am.

[ppat-0030102-b004] Schindler M, Assaf Y, Sharon N, Chipman DM (1977). Mechanism of lysozyme catalysis: Role of ground-state strain in subsite D in hen egg-white and human lysozymes. Biochemistry.

[ppat-0030102-b005] Blake CC, Johnson LN, Mair GA, North AC, Phillips DC (1967). Crystallographic studies of the activity of hen egg-white lysozyme. Proc R Soc London B Biol Sci.

[ppat-0030102-b006] Blundell J, Smith GJ, Perkins HR (1980). The peptidoglycan of Neisseria gonorrhoeae: *O*-acetyl groups and lysozyme sensitivity. FEMS Microbiol Lett.

[ppat-0030102-b007] Ghuysen JM, Strominger JL (1963). Structure of the cell wall of Staphylococcus aureus strain Copenhagen. II. Separation and structure of disaccharides. Biochemistry.

[ppat-0030102-b008] Martin HH, Gmeiner J (1979). Modification of peptidoglycan structure by penicillin-action in cell walls of Proteus mirabilis. Eur J Biochem.

[ppat-0030102-b009] Clarke AJ, Dupont C (1991). *O*-acetylated peptidoglycan: Its occurrence, pathobiological significance, and biosynthesis. Can J Microbiol.

[ppat-0030102-b010] Bera A, Herbert S, Jakob A, Vollmer W, Götz F (2005). Why are pathogenic staphylococci so lysozyme resistant? The peptidoglycan *O*-acetyltransferase OatA is the major determinant for lysozyme resistance of Staphylococcus aureus. Mol Microbiol.

[ppat-0030102-b011] Bera A, Biswas R, Herbert S, Götz F (2006). The presence of peptidoglycan *O*-acetyltransferase in various staphylococcal species correlates with lysozyme resistance and pathogenicity. Infect Immun.

[ppat-0030102-b012] Strominger JL, Ghuysen JM (1967). Mechanisms of enzymatic bacteriolysis. Cell walls of bacteria are solubilized by action of either specific carbohydrases or specific peptidases. Science.

[ppat-0030102-b013] Bera A, Biswas R, Herbert S, Kulauzovic E, Weidenmaier C (2007). Influence of wall teichoic acid on lysozyme resistance in Staphylococcus aureus. J Bacteriol.

[ppat-0030102-b014] Laible NJ, Germaine GR (1985). Bactericidal activity of human lysozyme, muramidase-inactive lysozyme, and cationic polypeptides against Streptococcus sanguis and Streptococcus faecalis: Inhibition by chitin oligosaccharides. Infect Immun.

[ppat-0030102-b015] During K, Porsch P, Mahn A, Brinkmann O, Gieffers W (1999). The non-enzymatic microbicidal activity of lysozymes. FEBS Lett.

[ppat-0030102-b016] Ibrahim HR, Matsuzaki T, Aoki T (2001). Genetic evidence that antibacterial activity of lysozyme is independent of its catalytic function. FEBS Lett.

[ppat-0030102-b017] Ibrahim HR, Thomas U, Pellegrini A (2001). A helix-loop-helix peptide at the upper lip of the active site cleft of lysozyme confers potent antimicrobial activity with membrane permeabilization action. J Biol Chem.

[ppat-0030102-b018] Kuroda M, Ohta T, Uchiyama I, Baba T, Yuzawa H (2001). Whole genome sequencing of meticillin-resistant Staphylococcus aureus. Lancet.

[ppat-0030102-b019] Cui L, Lian JQ, Neoh HM, Reyes E, Hiramatsu K (2005). DNA microarray-based identification of genes associated with glycopeptide resistance in Staphylococcus aureus. Antimicrob Agents Chemother.

[ppat-0030102-b020] Heilmann C, Hartleib J, Hussain MS, Peters G (2005). The multifunctional Staphylococcus aureus autolysin *aaa* mediates adherence to immobilized fibrinogen and fibronectin. Infect Immun.

[ppat-0030102-b021] Cramton SE, Gerke C, Schnell NF, Nichols WW, Götz F (1999). The intercellular adhesion (*ica*) locus is present in Staphylococcus aureus and is required for biofilm formation. Infect Immun.

[ppat-0030102-b022] Gerke C, Kraft A, Süssmuth R, Schweitzer O, Götz F (1998). Characterization of the *N*-acetylglucosaminyltransferase activity involved in the biosynthesis of the Staphylococcus epidermidis polysaccharide intercellular adhesin. J Biol Chem.

[ppat-0030102-b023] Heilmann C, Schweitzer O, Gerke C, Vanittanakom N, Mack D (1996). Molecular basis of intercellular adhesion in the biofilm-forming Staphylococcus epidermidis. Mol Microbiol.

[ppat-0030102-b024] Gross M, Cramton SE, Götz F, Peschel A (2001). Key role of teichoic acid net charge in Staphylococcus aureus colonization of artificial surfaces. Infect Immun.

[ppat-0030102-b025] Heilmann C, Hussain M, Peters G, Götz F (1997). Evidence for autolysin-mediated primary attachment of Staphylococcus epidermidis to a polystyrene surface. Mol Microbiol.

[ppat-0030102-b026] Peschel A, Otto M, Jack RW, Kalbacher H, Jung G (1999). Inactivation of the *dlt* operon in Staphylococcus aureus confers sensitivity to defensins, protegrins, and other antimicrobial peptides. J Biol Chem.

[ppat-0030102-b027] Bierbaum G, Sahl HG (1987). Autolytic system of Staphylococcus simulans 22: influence of cationic peptides on activity of *N*-acetylmuramoyl-*L*-alanine amidase. J Bacteriol.

[ppat-0030102-b028] Kawata S, Takemura T, Yokogawa K (1983). Characterization of two *N*-acetylmuramidases from Strepomyces globisporus 1829. Agric Biol Chem.

[ppat-0030102-b029] Endl J, Seidl HP, Fiedler F, Schleifer KH (1983). Chemical composition and structure of cell wall teichoic acids of staphylococci. Arch Microbiol.

[ppat-0030102-b030] Weidenmaier C, Kokai-Kun JF, Kristian SA, Chanturiya T, Kalbacher H (2004). Role of teichoic acids in Staphylococcus aureus nasal colonization, a major risk factor in nosocomial infections. Nat Med.

[ppat-0030102-b031] Ginsburg I (2004). Bactericidal cationic peptides can also function as bacteriolysis-inducing agents mimicking beta-lactam antibiotics? It is enigmatic why this concept is consistently disregarded. Med Hypotheses.

[ppat-0030102-b032] Ginsburg I (2001). Cationic peptides from leukocytes might kill bacteria by activating their autolytic enzymes causing bacteriolysis: Why are publications proposing this concept never acknowledged?. Blood.

[ppat-0030102-b033] Wecke J, Lahav M, Ginsburg I, Giesbrecht P (1982). Cell wall degradation of Staphylococcus aureus by lysozyme. Arch Microbiol.

[ppat-0030102-b034] Fischer W, Rosel P, Koch HU (1981). Effect of alanine ester substitution and other structural features of lipoteichoic acids on their inhibitory activity against autolysins of Staphylococcus aureus. J Bacteriol.

[ppat-0030102-b035] Cleveland RF, Wicken AJ, Daneo-Moore L, Shockman GD (1976). Inhibition of wall autolysis in Streptococcus faecalis by lipoteichoic acid and lipids. J Bacteriol.

[ppat-0030102-b036] Neuhaus FC, Baddiley J (2003). A continuum of anionic charge: Structures and functions of D-alanyl-teichoic acids in gram-positive bacteria. Microbiol Mol Biol Rev.

[ppat-0030102-b037] Steen A, Palumbo E, Deghorain M, Cocconcelli PS, Delcour J (2005). Autolysis of Lactococcus lactis is increased upon D-alanine depletion of peptidoglycan and lipoteichoic acids. J Bacteriol.

[ppat-0030102-b038] Meehl M, Herbert S, Götz F, Cheung A (2007). Interaction of the GraRS two-component system with the VraFG ABC-transporter to support vancomycin-intermediate resistance in Staphylococcus aureus. Antimicrob Agents Chemother.

[ppat-0030102-b039] Said-Salim B, Dunman PM, McAleese FM, Macapagal D, Murphy E (2003). Global regulation of Staphylococcus aureus genes by Rot. J Bacteriol.

[ppat-0030102-b040] Luong TT, Dunman PM, Murphy E, Projan SJ, Lee CY (2006). Transcription profiling of the *mgr*A regulon in Staphylococcus aureus. J Bacteriol.

[ppat-0030102-b041] Liang X, Zheng L, Landwehr C, Lunsford D, Holmes D (2005). Global regulation of gene expression by ArlRS, a two-component signal transduction regulatory system of Staphylococcus aureus. J Bacteriol.

[ppat-0030102-b042] Clarke AJ (1993). Extent of peptidoglycan *O* acetylation in the tribe *Proteeae*. J Bacteriol.

[ppat-0030102-b043] Kovacs M, Halfmann A, Fedtke I, Heintz M, Peschel A (2006). A functional *dlt* operon, encoding proteins required for incorporation of D-alanine in teichoic acids in gram-positive bacteria, confers resistance to cationic antimicrobial peptides in Streptococcus pneumoniae. J Bacteriol.

[ppat-0030102-b044] Goerke C, Fluckiger U, Steinhuber A, Bisanzio V, Ulrich M (2005). Role of Staphylococcus aureus global regulators sae and sigmaB in virulence gene expression during device-related infection. Infect Immun.

[ppat-0030102-b045] Resch A, Fehrenbacher B, Eisele K, Schaller M, Götz F (2005). Phage release from biofilm and planktonic Staphylococcus aureus cells. FEMS Microbiol Lett.

[ppat-0030102-b046] Kreiswirth BN, Lofdahl S, Betley MJ, O'Reilly M, Schlievert PM (1983). The toxic shock syndrome exotoxin structural gene is not detectably transmitted by a prophage. Nature.

[ppat-0030102-b047] Iordanescu S, Surdeanu M (1976). Two restriction and modification systems in Staphylococcus aureus NCTC8325. J Gen Microbiol.

[ppat-0030102-b048] Götz F (1990). Staphylococcus carnosus: A new host organism for gene cloning and protein production.. J Appl Bacteriol Symp Supp.

[ppat-0030102-b049] Hanahan D (1983). Studies on transformation of Escherichia coli with plasmids. J Mol Biol.

[ppat-0030102-b050] Brückner R (1997). Gene replacement in Staphylococcus carnosus and Staphylococcus xylosus. FEMS Microbiol Lett.

[ppat-0030102-b051] Peschel A, Ottenwälder B, Götz F (1996). Inducible production and cellular location of the epidermin biosynthetic enzyme EpiB using an improved staphylococcal expression system. FEMS Microbiol Lett.

[ppat-0030102-b052] Youngman P, Poth H, Green B, York K, Olmedo G, Smith I, Slepecky RA, Setlow P (1989). Methods for genetic manipulation, cloning and functional analysis of sporulation genes in Bacillus subtilis. Regulation of procaryotic development.

